# Supplementary material for: Powerline right-of-way management and flower-visiting insects: How vegetation management can promote pollinator diversity
Source: PLoS One. 2021 Jan 6;16(1):e0245146. doi: 10.1371/journal.pone.0245146 (PMC7787533; doi:10.1371/journal.pone.0245146)
Supplement: S1 Appendix — (DOCX) [file pone.0245146.s001.docx]

**S1 Appendix** Powerline right-of-way management and flower-visiting insects: How vegetation management can promote pollinator diversity

L. Russo, H. Stout, D. Roberts, B. Ross, C. Mahan

**S1 Table**. A list of the treatment sites, their size, and the number of litres of herbicide applied per hectare.

| **Plot Code** | **Ha** | **Litres/Ha 2016** | **2016 Application** | **Herbicides Used** | **Plant Species Richness** | **Bee Species Richness** | **Bee Abundance** | **Non-bee Morphospecies Richness^d^** | **Non-bee Abundance^d^** |
| --- | --- | --- | --- | --- | --- | --- | --- | --- | --- |
| BLV3 | 1.12 | 9.74 | Low Volume Basal | Aminopyralid, Imazapyr, Triclopyr^a^ | 30 | 76 | 652 | 53 | 155 |
| F2 | 1.14 | 249.66 | High Volume Foliar | Aminopyralid, Imazapyr, Triclopyr, Picloram, Glyphosate^b^ | 17 | 57 | 329 | 55 | 135 |
| HC1 | 1.19 | 0 | Hand Cut Only | NA | 18 | 40 | 115 | 46 | 63 |
| MH1 | 0.81 | 2.32 | Low Volume Foliar | Glyphosate, Imazapyr^c^ | 16 | 69 | 303 | 50 | 172 |
| MH2 | 0.81 | 9.31 | Low Volume Foliar | Glyphosate, Imazapyr^c^ | 27 | 78 | 491 | 50 | 102 |
| SF2 | 1.34 | 70.65 | High Volume Foliar | Aminopyralid, Imazapyr, Triclopyr, Picloram, Glyphosate^b^ | 16 | 61 | 454 | 54 | 116 |

^a^Garlon 3A (triclopyr) 5 pints/100 gal + Milestone (aminopyralid) 7 oz/100 gal + Arsenal (imazapyr) 1%

^b^Milestone (aminopyralid) 7 oz/100 gal + Rodeo (glyphosate) 1% + Arsenal (imazapyr) 1% + Tordon K (picloram) 4% + Garlon 3A (triclopyr) 5 pints/100 gal

^c^Accord concentrate (glyphosate) 7% + Arsenal (imazapyr) 1%

^d^2016 only

**S2 Table**. A list of all bee and non-bee species collected in the bowl survey conducted in 2016, in three different colored bowls (blue, yellow, and white). Species in blue are cleptoparasitic (cuckoo bees).

| **BEE TAXA** | **Blue** | **Yellow** | **White** |
| --- | --- | --- | --- |
| **ANDRENIDAE** (mining bees) |  |  |  |
| ***Calliopsis andreniformis*** |  | 1 |  |
| **APIDAE** (cuckoo/carpenter/digger bees, bumble bees and honey bees) |  |  |  |
| ***Ceratina* sp.** (small carpenter bee) | 1 |  |  |
| ***Ceratina dupla*** (doubled ceratina) | 7 |  | 2 |
| ***Ceratina strenua*** (nimble ceratina) | 1 |  |  |
| ***Holcopasites* sp.** (cuckoo bee) |  |  | 1 |
| ***Melissodes trinodis*** | 1 |  |  |
| ***Peponapis pruinosa*** (squash bee, Eastern cucurbit bee) | 1 |  |  |
| **COLLETIDAE** (plasterer bees, masked/yellow-faced bees) |  |  |  |
| ***Hylaeus affinis/modestus*** |  | 2 |  |
| **HALICTIDAE** (sweat bees) |  |  |  |
| ***Augochlorella aurata*** | 2 | 2 |  |
| ***Halictus ligatus*** | 3 |  |  |
| ***Lasioglossum abanci*** | 1 |  |  |
| ***Lasioglossum coriaceum*** | 1 |  |  |
| ***Lasioglossum cressonii*** | 1 |  | 1 |
| ***Lasioglossum ephialtum*** | 2 |  |  |
| ***Lasioglossum heterognathum*** |  |  | 1 |
| ***Lasioglossum tegulare*** | 1 |  |  |
| **MEGACHILIDAE** (leaf-cutter bees, mason bees) |  |  |  |
| ***Hoplitis producta*** | 1 |  |  |
| ***Megachile pugnata*** (pugnacious leaf-cutter bee) | 1 |  |  |
| ***Osmia atriventris*** (Maine blueberry bee) | 1 | 1 |  |
| **NON-BEE TAXA** | **Blue** | **Yellow** | **White** |
| **Coleoptera** (beetles) |  |  |  |
| **Chrysomelidae** | 1 |  | 1 |
| **Mordellidae** (tumbling flower beetles) | 1 |  |  |
| **Diptera** (true flies) | 2 |  |  |
| **Dolichopodidae** (long-legged flies) |  | 1 | 1 |
| **Sarcophagidae** (flesh flies) |  | 1 | 2 |
| **Syrphidae** (hover flies, flower flies) | 1 |  |  |
| **Tachinidae** (tachinid flies) |  | 1 |  |
| **Tephritidae** (fruit flies) |  |  |  |
| *Eutreta* sp. |  | 1 |  |
| **Hemiptera** (true bugs) |  |  |  |
| **Fulgoroidea** (planthoppers) |  | 1 |  |
| **Hymenoptera** (wasps and ants) |  |  |  |
| **-Orgilinae** |  | 2 |  |
| **-Rogadinae** | 1 |  |  |
| **Chrysididae** (cuckoo wasps) |  |  |  |
| *Elampus* sp. |  | 2 | 1 |
| **Crabronidae** (square-headed wasps) |  |  |  |
| *Nysson* sp. |  | 1 |  |
| **Cynipidae** (gall wasps) |  | 1 |  |
| **Diapriidae** |  | 7 | 2 |
| **-Ichneumoninae** |  | 2 |  |
| **-Phygadeuontinae** |  | 2 | 1 |
| **Pompilidae** (spider wasps) |  |  |  |
| *Aporinellus* sp. | 2 |  | 2 |
| *Arachnospila* sp. |  | 1 |  |
| *Minagenia* sp. |  |  | 1 |
| **Pteromalidae** | 1 | 1 |  |
| **Scelionidae** | 1 |  |  |
| **Tenthredinidae** (common sawflies) |  | 2 |  |

**S3 Table**. Presence/absence of the plant species recorded in the 2016 survey.

| Latin Name | Hand Cut (HC1) | Low Volume Foliar (MH1) | Low Volume Foliar (MH3) | Low Volume Basal (BLV3) | High Volume Foliar (SF2) | High Volume Foliar (F2) | Sum |
| --- | --- | --- | --- | --- | --- | --- | --- |
| *Achillea millefolium* | 0 | 0 | 1 | 0 | 0 | 0 | 1 |
| *Apocynum cannabinum* | 0 | 0 | 0 | 1 | 0 | 0 | 1 |
| *Cirsium sp.* | 0 | 0 | 1 | 0 | 0 | 0 | 1 |
| *Corylus sp.* | 0 | 0 | 0 | 1 | 0 | 0 | 1 |
| *Disporum sp.* | 0 | 0 | 0 | 1 | 0 | 0 | 1 |
| *Erigeron sp.* | 0 | 0 | 0 | 1 | 0 | 0 | 1 |
| *Euphorbia sp.* | 1 | 0 | 0 | 0 | 0 | 0 | 1 |
| Fabaceae | 0 | 0 | 0 | 0 | 1 | 0 | 1 |
| *Hieracium sp.* | 0 | 0 | 1 | 0 | 0 | 0 | 1 |
| *Kalmia latifolia* | 0 | 0 | 0 | 1 | 0 | 0 | 1 |
| *Lonicera sp.* | 0 | 0 | 0 | 1 | 0 | 0 | 1 |
| *Maianthemum racemosum* | 0 | 0 | 0 | 1 | 0 | 0 | 1 |
| *Mentha sp.* | 0 | 0 | 0 | 1 | 0 | 0 | 1 |
| *Potentilla sp.* | 1 | 0 | 0 | 0 | 0 | 0 | 1 |
| *Prunus virginiana* | 0 | 1 | 0 | 0 | 0 | 0 | 1 |
| *Rhododendron* | 0 | 0 | 0 | 1 | 0 | 0 | 1 |
| *Sambucus sp.* | 0 | 0 | 0 | 0 | 0 | 1 | 1 |
| *Spirea sp.* | 0 | 0 | 1 | 0 | 0 | 0 | 1 |
| *Trientalis borealis* | 0 | 0 | 1 | 0 | 0 | 0 | 1 |
| *Vicia sp.* | 0 | 0 | 0 | 1 | 0 | 0 | 1 |
| *Vitis sp.* | 1 | 0 | 0 | 0 | 0 | 0 | 1 |
| *Asclepias tuberosa* | 0 | 0 | 1 | 1 | 0 | 0 | 2 |
| *Bellis perennis* | 0 | 0 | 1 | 1 | 0 | 0 | 2 |
| *Centaurea calcitrapa* | 0 | 0 | 1 | 1 | 0 | 0 | 2 |
| Cyperaceae | 0 | 0 | 1 | 0 | 0 | 1 | 2 |
| *Dentaria diphylla* | 1 | 0 | 1 | 0 | 0 | 0 | 2 |
| *Hypericum* | 0 | 1 | 0 | 1 | 0 | 0 | 2 |
| *Sicyos angulatus* | 0 | 1 | 0 | 1 | 0 | 0 | 2 |
| *Smilax sp.* | 0 | 0 | 1 | 0 | 1 | 0 | 2 |
| *Trillium sp.* | 0 | 0 | 1 | 0 | 0 | 1 | 2 |
| *Viburnum acerifolium* | 0 | 0 | 0 | 0 | 1 | 1 | 2 |
| *Viola sp.* | 0 | 0 | 1 | 0 | 0 | 1 | 2 |
| *Fragaria sp.* | 0 | 0 | 1 | 1 | 1 | 0 | 3 |
| *Quercus berberidifolia* | 1 | 1 | 0 | 1 | 0 | 0 | 3 |
| *Ribes sp.* | 1 | 0 | 0 | 1 | 0 | 1 | 3 |
| *Rumex acetosella* | 0 | 0 | 1 | 0 | 1 | 1 | 3 |
| *Uvularia sp.* | 1 | 0 | 1 | 0 | 0 | 1 | 3 |
| *Aralia nudicaulis* | 1 | 1 | 1 | 1 | 0 | 0 | 4 |
| *Vaccinium corymbosum* | 1 | 1 | 0 | 1 | 1 | 0 | 4 |
| *Comptonia peregrina* | 0 | 1 | 1 | 1 | 1 | 1 | 5 |
| *Vaccinium angustifolium* | 1 | 1 | 1 | 1 | 1 | 0 | 5 |
| *Dennstaedtia punctilobula* | 1 | 1 | 1 | 1 | 1 | 1 | 6 |
| *Gaultheria procumbens* | 1 | 1 | 1 | 1 | 1 | 1 | 6 |
| *Hamamelis virginiana* | 1 | 1 | 1 | 1 | 1 | 1 | 6 |
| *Lysimachia quadrifolia* | 1 | 1 | 1 | 1 | 1 | 1 | 6 |
| Poaceae | 1 | 1 | 1 | 1 | 1 | 1 | 6 |
| *Pteridium sp.* | 1 | 1 | 1 | 1 | 1 | 1 | 6 |
| *Rubus sp.* | 1 | 1 | 1 | 1 | 1 | 1 | 6 |
| *Solidago sp.* | 1 | 1 | 1 | 1 | 1 | 1 | 6 |
|  | 18 | 16 | 27 | 30 | 16 | 17 |  |

**S4 Table.** Table of bee species collected across both years via bowl and net sampling.

| **NUMBER OF INDIVIDUAL BEES PER ROW SITE** | | | |
| --- | --- | --- | --- |
|  | **SGL33 (2016)** | **SGL33 (2016)** | **SGL33 (2017)** |
|  | *(1 bowl plot)* | *(6 net plots)* | *(6 net plots)* |
| **BEE TAXA** | *(24* ***bowl*** *hours/plot)* | *(16* ***net*** *hours/plot)* | *(16* ***net*** *hours/plot)* |
| **ANDRENIDAE** (mining bees) |  |  |  |
| ***Andrena* sp.** |  | 28 |  |
| ***Andrena bradleyi*** ("Bradley's mining bee") |  |  | 1 |
| ***Andrena brevipalpis*** |  | 2 |  |
| ***Andrena brevipalpis/robertsonii*** |  |  |  |
| ***Andrena carlini*** ("Carlin's mining bee") |  | 11 | 14 |
| ***Andrena carolina*** ("Carolina mining bee") |  | 18 | 18 |
| ***Andrena ceanothi*** |  | 8 | 17 |
| ***Andrena commoda*** |  |  |  |
| ***Andrena crataegi*** ("Hawthorn mining bee") |  | 2 |  |
| ***Andrena cressonii*** ("Cresson's mining bee") |  | 1 |  |
| ***Andrena forbesii*** ("Forbes' mining bee") |  |  | 1 |
| ***Andrena hirticincta*** ("hairy-banded mining bee") |  |  | 5 |
| ***Andrena imitatrix*** |  | 15 | 1 |
| ***Andrena krigiana*** ("dwarf-dandelion mining bee") |  |  |  |
| ***Andrena mandibularis*** |  | 5 |  |
| ***Andrena milwaukeensis*** ("Milwaukee mining bee") |  | 1 | 2 |
| ***Andrena miserabilis*** ("miserable mining bee") |  | 1 |  |
| ***Andrena nasonii*** ("Nason's mining bee") |  |  | 1 |
| ***Andrena nivalis*** ("snowy mining bee") |  | 27 | 7 |
| ***Andrena nubecula*** ("cloudy-winged mining bee") |  | 1 | 1 |
| ***Andrena personata*** |  | 3 |  |
| ***Andrena pruni*** ("cherry mining bee") |  |  |  |
| ***Andrena robertsonii*** |  |  |  |
| ***Andrena rugosa*** ("rugose mining bee") |  | 2 |  |
| ***Andrena sayi*** ("Say's mining bee") |  |  |  |
| ***Andrena spiraeana*** |  | 4 | 5 |
| ***Andrena tridens*** |  | 1 |  |
| ***Andrena vicina*** ("neighborly mining bee") |  | 10 | 12 |
| ***Andrena virginiana*** ("Virginia mining bee") |  | 31 | 55 |
| ****Andrena wilkella*** ("Wilke's mining bee") |  | 9 | 10 |
| ***Andrena ziziaeformis*** |  |  | 6 |
| ***Andrena* (Trachandrena) sp.** (males) |  |  | 5 |
| ***Calliopsis andreniformis*** | 1 |  |  |
| **APIDAE** (cuckoo/carpenter/digger bees, bumble bees and honey bees) |  |  |  |
| ***Anthophora* sp.** (long-horned digger bees) |  |  |  |
| ***Anthophora abrupta*** ("abrupt digger bee") |  |  |  |
| ***Anthophora bomboides*** ("bumble-bee digger bee") |  |  |  |
| ****Apis mellifera*** ("European honey bee") |  | 226 | 20 |
| ***Bombus* sp.** (bumble bees) |  | 3 |  |
| ***Bombus bimaculatus*** ("two-spotted bumble bee") |  | 16 | 91 |
| ***Bombus fernaldae*** ("Fernald's bumble bee", social parasite of other *Bombus* sp.) |  | 1 |  |
| ***^1^Bombus fervidus*** ("yellow bumble bee") |  | 1 | 2 |
| ***Bombus griseocollis*** ("brown-belted bumble bee") |  | 3 | 3 |
| ***Bombus impatiens*** ("common Eastern bumble bee") |  | 65 | 276 |
| ***Bombus perplexus*** ("confusing/perplexing bumble bee") |  |  | 2 |
| ***^2^Bombus sandersoni*** ("Sanderson's bumble bee") |  | 1 | 1 |
| ***Bombus vagans*** ("half-black bumble bee") |  | 23 | 48 |
| ***Ceratina* sp.** (small carpenter bees) | 1 | 11 | 16 |
| ***Ceratina calcarata*** ("spurred small carpenter bee") |  | 9 | 20 |
| ***Ceratina dupla*** ("doubled small carpenter bee") | 9 | 57 | 120 |
| ***Ceratina miqmaki*** ("Miqmak small carpenter bee") |  | 10 | 28 |
| ***Ceratina strenua*** ("nimble small carpenter bee") | 1 | 4 | 16 |
| ***Epeolus scutellaris*** ("notch-backed cellophane-cuckoo bee", host: *Colletes simulans armatus*) |  | 2 | 3 |
| ***Holcopasites calliopsidis*** (cleptoparasite of *Calliopsis andreniformis*) | 1 | 1 |  |
| ***Melissodes* sp.** (longhorned bees) |  | 1 | 1 |
| ***^3^Melissodes apicatus*** |  |  |  |
| ***Melissodes druriellus*** |  |  | 1 |
| ***Melissodes illatus/subillatus*** |  |  | 3 |
| ***Melissodes trinodis*** | 1 | 2 |  |
| **Nomadinae** |  |  |  |
| ***Nomada* sp.** (nomad bees, hosts: usually *Andrena* sp.) |  |  |  |
| ***Nomada bidentata* group** |  | 6 | 4 |
| ***Nomada cressonii*** ("Cresson's nomad bee") |  | 5 |  |
| ***Nomada denticulata*** |  |  | 1 |
| ***Nomada imbricata*** |  |  | 1 |
| ***Nomada luteoloides*** |  | 2 | 1 |
| ***Nomada maculata*** ("spotted nomad bee") |  | 3 | 7 |
| ***Nomada pygmaea*** ("pygmy nomad bee") |  | 8 | 6 |
| ***Nomada sayi/illinoensis*** |  |  |  |
| ***Nomada vicina*** |  |  | 2 |
| ***^3^Nomada xanthura*** |  |  | 1 |
| ***Peponapis pruinosa*** ("Eastern cucurbit bee") | 1 |  |  |
| ***Triepeolus donatus*** ("thistle longhorn-cuckoo bee", host: *Melissodes desponsa*) |  |  | 1 |
| ***Xylocopa virginica*** ("Eastern/large carpenter bee") |  | 6 | 6 |
| **COLLETIDAE** (plasterer bees, masked/yellow-faced bees) |  |  |  |
| ***Colletes* sp.** (cellophane/polyester bees) |  |  |  |
| ***Colletes simulans*** ("spine-shouldered cellophane bee") |  | 7 | 6 |
| ***Colletes thoracicus*** ("rufous-chested cellophane bee") |  |  |  |
| ***Colletes validus*** ("blueberry cellophane bee") |  | 1 |  |
| ***Hylaeus* sp.** (masked bees) |  |  |  |
| ***Hylaeus affinis*** ("Eastern masked bee") |  | 5 | 2 |
| ***Hylaeus affinis/modestus*** ("Eastern/modest masked bee") | 2 | 46 | 55 |
| ***Hylaeus annulatus*** ("annulate masked bee") |  | 6 | 4 |
| ***Hylaeus mesillae*** ("Mesilla masked bee") |  | 13 | 22 |
| ***Hylaeus modestus*** ("modest masked bee") |  | 19 | 15 |
| **HALICTIDAE** (sweat bees) |  |  |  |
| ***Agapostemon virescens*** ("bicolored striped sweat bee") |  |  |  |
| ***Augochlora pura*** ("pure green sweat bee") |  | 7 | 14 |
| ***Augochlorella aurata*** | 4 | 21 | 31 |
| ***Augochloropsis* sp.** (metallic green sweat bees) |  |  |  |
| ***Augochloropsis metallica*** |  | 1 |  |
| ***Augochloropsis metallica fulgida*** |  | 10 | 17 |
| ***Halictus* sp.** (furrow bees) |  |  |  |
| ***Halictus confusus*** ("confusing metallic furrow bee") |  | 20 | 10 |
| ***Halictus ligatus*** ("ligated furrow bee") | 3 | 41 | 23 |
| ***Halictus rubicundus*** ("orange-legged furrow bee") |  | 2 | 3 |
| ***Lasioglossum* sp.** |  | 16 | 5 |
| ***Lasioglossum abanci*** | 1 | 2 |  |
| ***Lasioglossum acuminatum*** |  |  | 1 |
| ***Lasioglossum albipenne*** ("white-winged metallic sweat bee") |  |  | 1 |
| ***Lasioglossum apocyni*** |  | 1 | 12 |
| ***Lasioglossum cinctipes*** |  |  | 1 |
| ***Lasioglossum coeruleum*** |  |  |  |
| ***Lasioglossum coriaceum*** | 1 | 7 | 5 |
| ***Lasioglossum cressonii*** ("Cresson's Dialictus sweat bee") | 2 | 25 | 73 |
| ***Lasioglossum ephialtum*** | 2 | 4 | 3 |
| ***Lasioglossum foxii*** |  | 2 | 1 |
| ***Lasioglossum fuscipenne*** ("dark-winged sweat bee") |  |  |  |
| ***Lasioglossum heterognathum*** | 1 | 28 | 36 |
| ***Lasioglossum hitchensi*** |  | 1 | 5 |
| ***Lasioglossum illinoense*** |  |  |  |
| ***Lasioglossum imitatum*** |  | 2 |  |
| ***Lasioglossum laevissimum*** |  |  | 1 |
| ***Lasioglossum leucocomum*** |  |  | 1 |
| ****Lasioglossum leucozonium*** ("white-zoned mining/furrow bee") |  | 1 | 1 |
| ***Lasioglossum lineatulum*** ("lineated metallic sweat bee") |  | 13 | 2 |
| ***Lasioglossum nigroviride*** ("black and green Dialictus sweat bee") |  |  | 2 |
| ***Lasioglossum paradmirandum*** |  | 1 |  |
| ***Lasioglossum perpunctatum*** |  |  | 1 |
| ***Lasioglossum quebecense*** |  | 4 | 1 |
| ***Lasioglossum subviridatum*** |  |  | 1 |
| ***Lasioglossum tegulare*** ("epaulette metallic sweat bee") | 1 |  |  |
| ***Lasioglossum timothyi*** |  |  | 1 |
| ***Lasioglossum trigeminum*** |  |  | 2 |
| ***Lasioglossum truncatum*** |  | 1 | 1 |
| ***Lasioglossum versans*** |  | 11 | 15 |
| ***Lasioglossum versatum*** |  |  | 2 |
| ****Lasioglossum zonulum*** |  |  | 1 |
| ***Sphecodes* sp.** ("blood bees", hosts: usually other Halictids) |  | 4 | 6 |
| ***Sphecodes coronus*** |  |  | 1 |
| ***Sphecodes galerus*** |  |  | 2 |
| ***Sphecodes heraclei*** |  |  | 2 |
| **MEGACHILIDAE** (leaf-cutter and mason bees) |  |  |  |
| ***Coelioxys* sp.** (cuckoo leaf-cutter bees, hosts: usually *Megachile* sp.) |  |  |  |
| ***Coelioxys modesta/modestus*** ("modest cuckoo leafcutter bee") |  |  | 1 |
| ***Coelioxys moesta/moestus*** |  |  | 1 |
| ***Coelioxys octodentata*** ("eight-toothed cuckoo leaf-cutter bee") |  | 1 |  |
| ***Coelioxys rufitarsis*** ("red-footed cuckoo leaf-cutter bee") |  | 1 | 2 |
| ***Coelioxys sayi*** ("Say's cuckoo leaf-cutter bee") |  | 1 | 1 |
| ***Heriades* sp.** |  |  |  |
| ***Heriades carinata*** |  |  | 6 |
| ***^3^Heriades leavitti*** |  | 1 | 2 |
| ***Heriades leavitti/variolosa*** |  | 1 |  |
| ***Hoplitis* sp.** |  |  |  |
| ***Hoplitis pilosifrons*** |  | 1 | 3 |
| ***Hoplitis producta*** | 1 |  | 5 |
| ***Hoplitis spoilata*** |  |  | 1 |
| ***Megachile* sp.** (leafcutter/resin bees) |  |  |  |
| ***Megachile brevis*** ("common little leafcutter bee") |  |  |  |
| ***Megachile campanulae*** ("bellflower resin bee") |  | 1 | 1 |
| ***Megachile gemula*** ("small-handed leafcutter bee") |  | 4 | 2 |
| ***Megachile inermis*** ("unarmed leafcutter bee") |  | 1 | 1 |
| ***Megachile inimica*** ("hostile leafcutter bee") |  |  | 1 |
| ***Megachile latimanus*** ("broad-handed leafcutter bee") |  | 11 | 3 |
| ***Megachile mendica*** ("flat-tailed leafcutter bee") |  | 15 | 5 |
| ***Megachile montivaga*** ("silver-tailed petalcutter bee") |  |  | 3 |
| ***Megachile pugnata*** ("pugnacious/sunflower leafcutter bee") | 1 | 3 | 1 |
| ***Megachile relativa*** ("golden-tailed leafcutter bee") |  | 29 | 6 |
| ****Megachile sculpturalis*** ("giant/sculptured resin bee") |  |  | 1 |
| ***Osmia* sp.** (mason bees) |  |  |  |
| ***Osmia atriventris*** ("Maine blueberry bee") | 2 | 3 | 6 |
| ***Osmia bucephala*** ("bufflehead mason bee") |  | 1 | 1 |
| ***Osmia collinsiae*** |  |  | 1 |
| ****Osmia cornifrons*** ("(Japanese) hornfaced bee") |  | 8 |  |
| ***Osmia distincta*** |  |  |  |
| ***Osmia georgica*** ("Georgia mason bee") |  |  | 1 |
| ***Osmia inspergens*** |  | 2 |  |
| ****Osmia lignaria*** ("blue/mason orchard bee") |  |  |  |
| ***Osmia pumila*** |  | 1 | 2 |
| ****Osmia taurus*** ("taurus/bull mason bee") |  | 2 |  |
| **^2^MELITTIDAE** (oil-collecting bees) |  |  |  |
| ***Macropis ciliata*** ("ciliary oil-collecting bee") |  | 1 | 1 |
| **TOTAL INDIVIDUAL BEES PER SITE** | **36** | **1056** | **1288** |
| **TOTAL BEE TAXA PER SITE** | **19** | **95** | **110** |

*Indicates non-native species. ^1^Vulnerable species. ^2^Uncommon species. ^3^State record.

**S5 Table**. Pearson correlations between measured attributes at the five research sites (excluding the hand-cut site) across 2016 and 2017.

| **Comparison** | **Pearson's product-moment correlation** | **P value** |
| --- | --- | --- |
| Bee abundance and bee species richness | 0.66 | << 0.001 |
| Litres/ha and plant species richness | -0.48 | << 0.001 |
| Bee abundance and Litres/ha | -0.20 | 0.09 |
| Bee abundance and plant species richness | 0.30 | 0.008 |
| Bee species richness and Litres/ha | -0.27 | 0.02 |
| Bee species richness and plant species richness | 0.22 | 0.06 |
| Non-bee abundance and morphospecies richness | 0.60 | < 0.001 |
| Non-bee abundance and Litres/ha | -0.05 | 0.76 |
| Non-bee abundance and plant species richness | -0.05 | 0.74 |
| Non-bee morphospecies richness and Litres/ha | 0.02 | 0.91 |
| Non-bee morphospecies richness and plant species richness | -0.10 | 0.56 |

**S6 Table.** Results of GLMMs of treatment effects on bee abundance after removing *B. impatiens* and *A. mellifera* from the analysis*.*

| **Response Variable** | **Fixed Effect** | **Contrast** | **Random Effect** | **Effect Size** | **t value** | **p value** | **AIC** |
| --- | --- | --- | --- | --- | --- | --- | --- |
| Log(Abundance - B. impatiens and A. mellifera) | 2012 application (continuous) | NA | Year, Time | < 0.001 | 0.83 | 0.41 | 222.96 |
|  | 2012 application (categorical) | HVF - LVF | Year, Time | -0.22 | -1.09 | 0.28 | 222.47 |
|  | 2016 application (continuous) | NA | Year, Time | < 0.001 | -0.06 | 0.95 | 223.65 |
|  | 2016 application (categorical) | LVB - HVF | Year, Time | 0.01 | 0.02 | 0.98 | 225.41 |
|  |  | LVF - HVF | Year, Time | 0.23 | 51 | 0.61 |  |
|  |  | LVF - LVB | Year, Time | 0.22 | 0.39 | 0.69 |  |
|  | 2016 plant species richness | NA | Year, Time | 0.02 | 1.33 | 0.19 | 225.41 |

**S7 Table**. Results of a rarefaction analysis comparing categorical treatments

| **Treatment Application** | **Diversity Index** | **Observed** | **Estimator** | **Standard Error** | **Lower Confidence Interval** | **Upper Confidence Interval** |
| --- | --- | --- | --- | --- | --- | --- |
| HVF | Shannon diversity | 31.4 | 33.66 | 1.46 | 31.4 | 36.52 |
| LVB | Shannon diversity | 18.18 | 19.98 | 1.34 | 18.18 | 22.6 |
| LVF | Shannon diversity | 43.53 | 47.9 | 2.19 | 43.6 | 52.19 |
|  |  |  |  |  |  |  |
| HVF | Simpson diversity | 19.48 | 19.95 | 0.98 | 19.48 | 21.86 |
| LVB | Simpson diversity | 7.07 | 7.13 | 0.57 | 7.07 | 8.25 |
| LVF | Simpson diversity | 24.37 | 25.11 | 1.52 | 24.37 | 28.09 |
|  |  |  |  |  |  |  |
| HVF | Species richness | 79 | 99.32 | 11.13 | 86.45 | 134.46 |
| LVB | Species richness | 76 | 113.44 | 18.47 | 91 | 169.46 |
| LVF | Species richness | 101 | 139.49 | 17.65 | 117.35 | 191.61 |


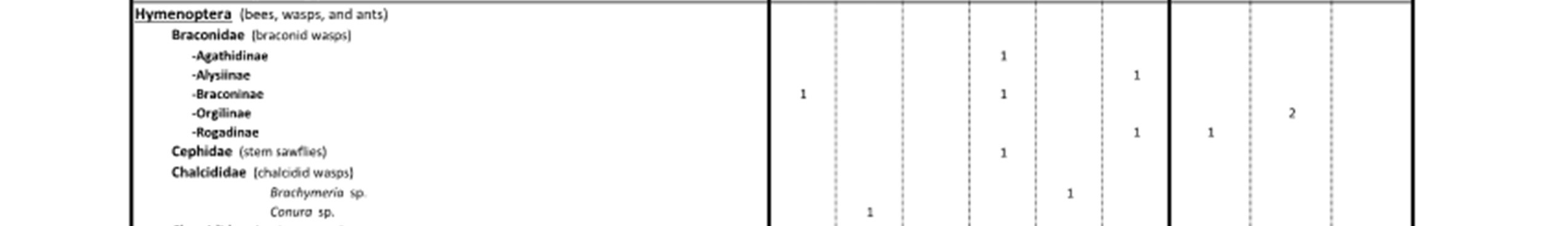

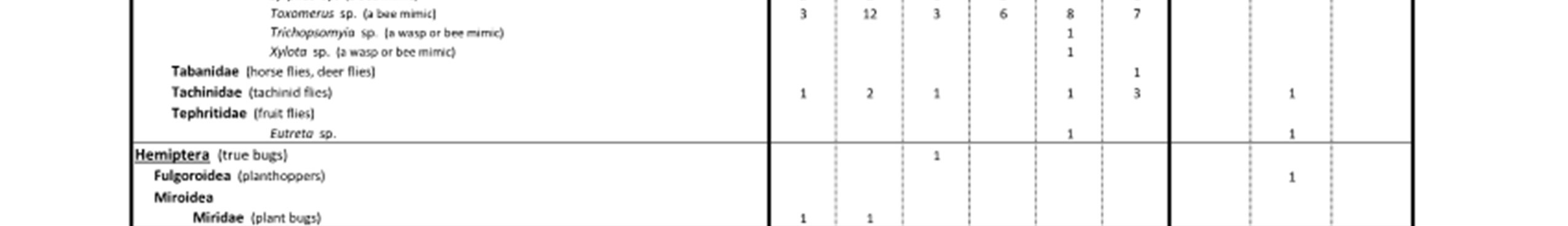

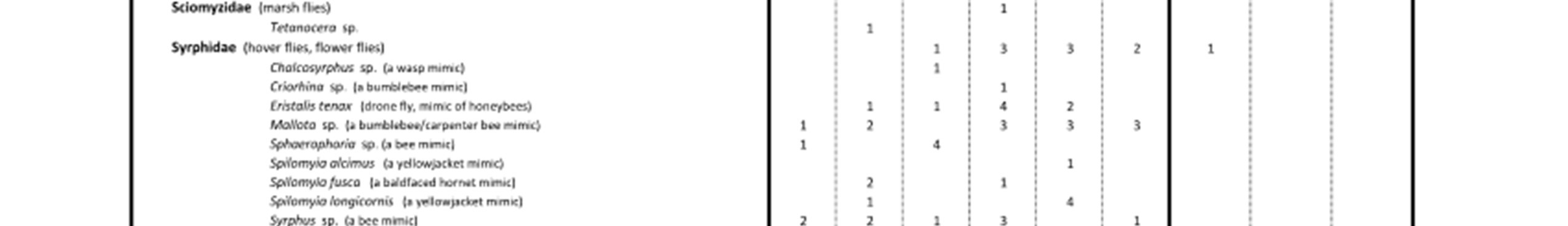

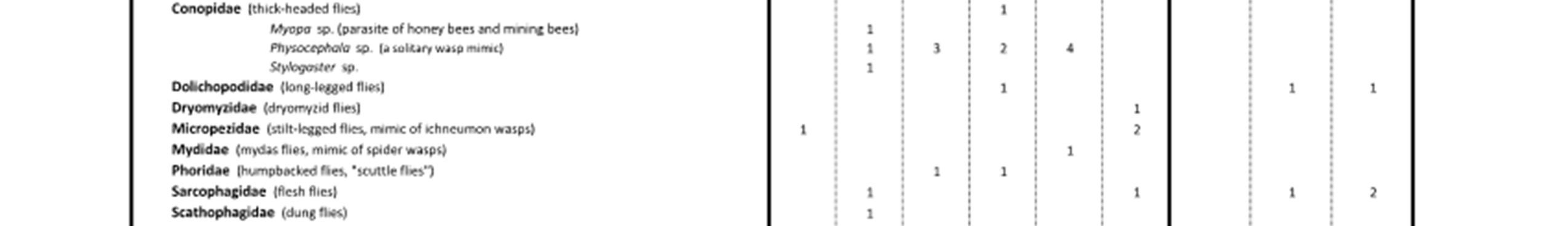

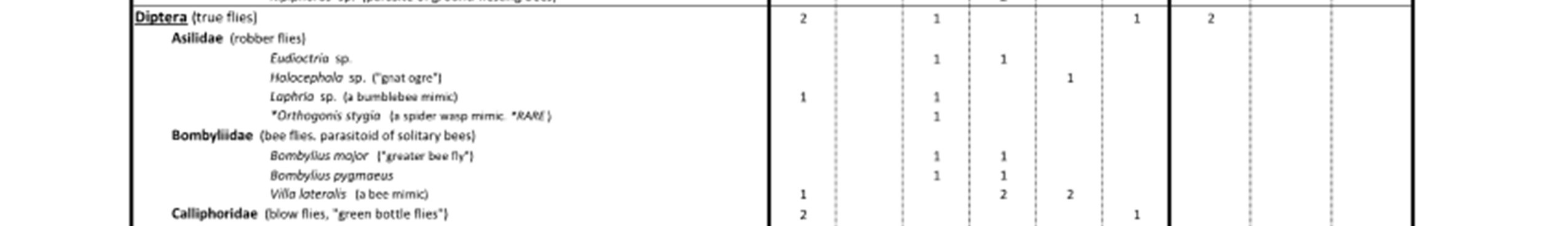

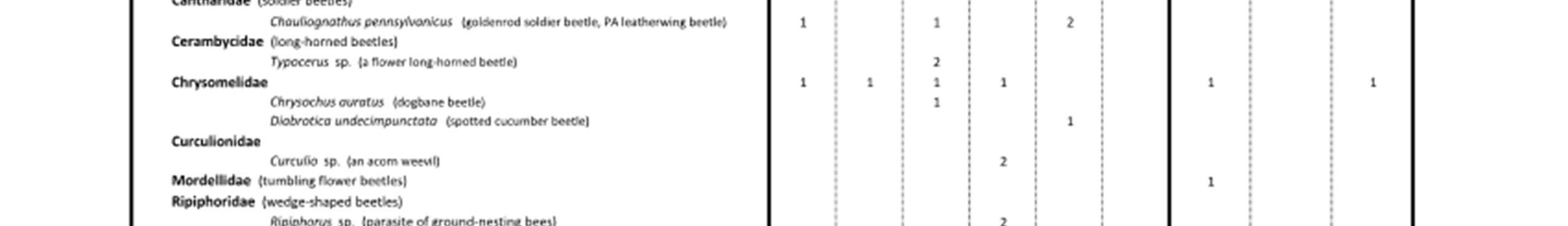

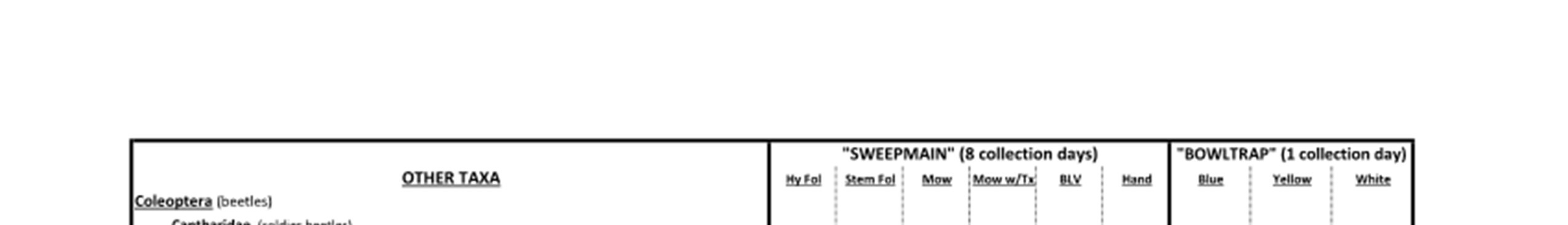
**S8 Table.** Non-bee morphospecies collected in 2016 via bowl and net sampling.

**
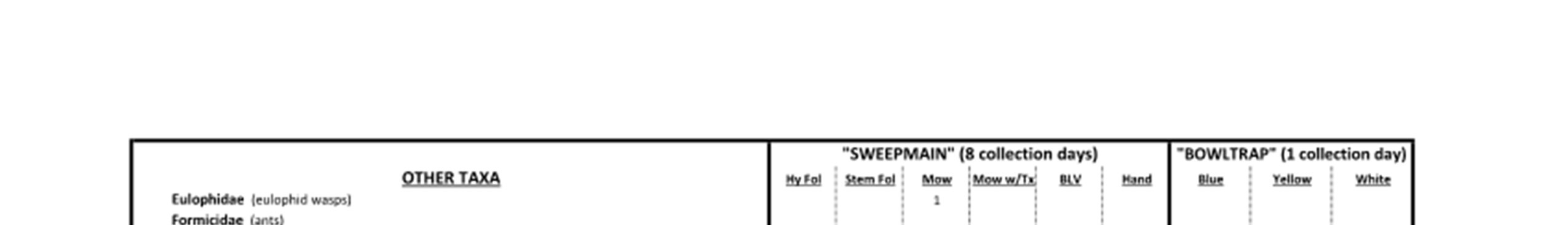

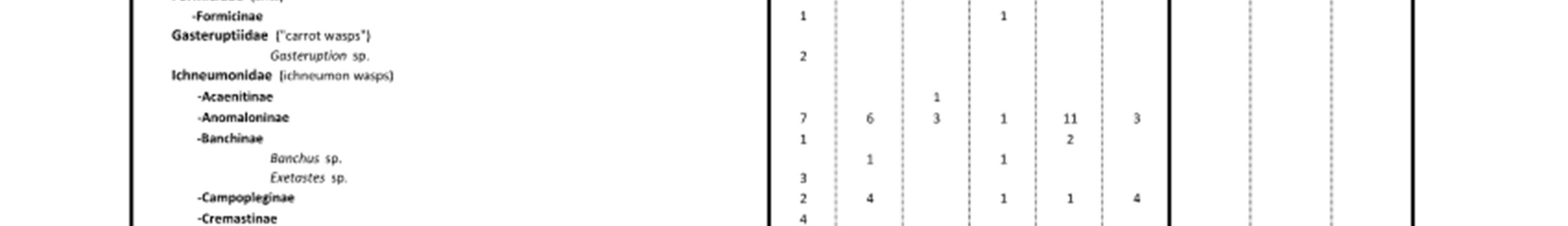
**
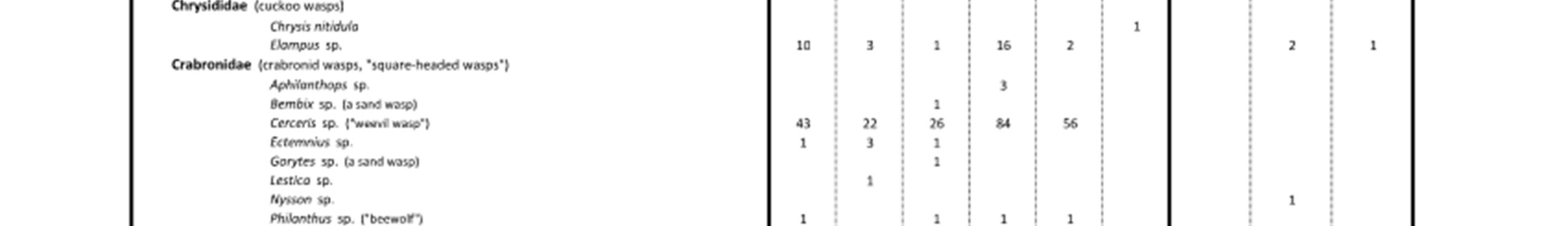

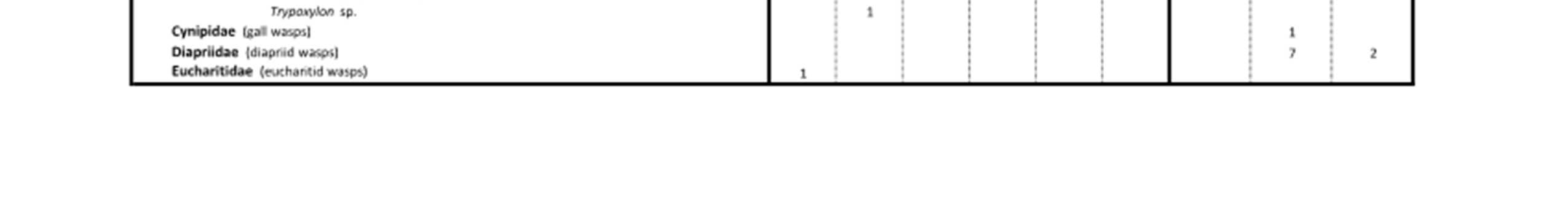
**
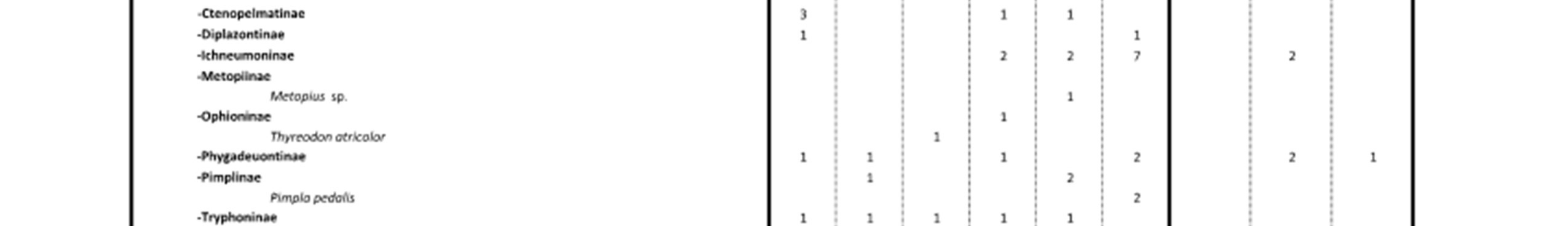

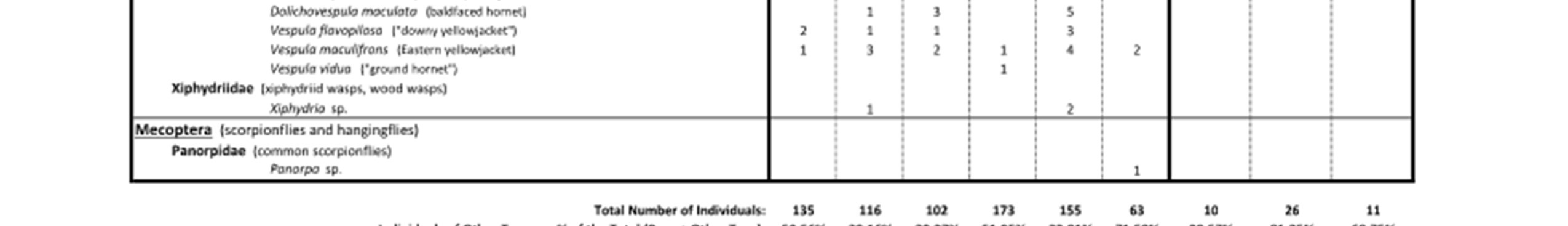

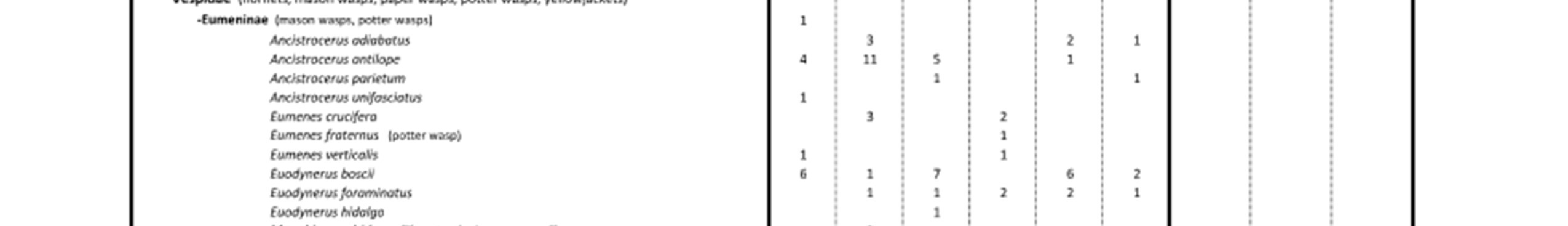

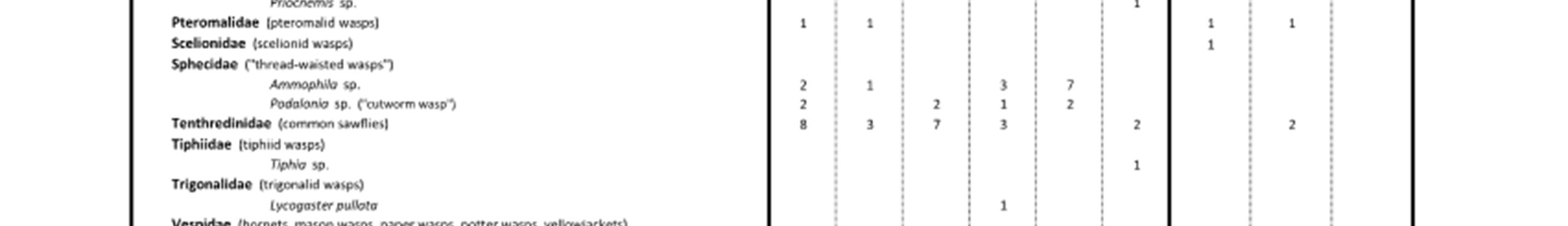

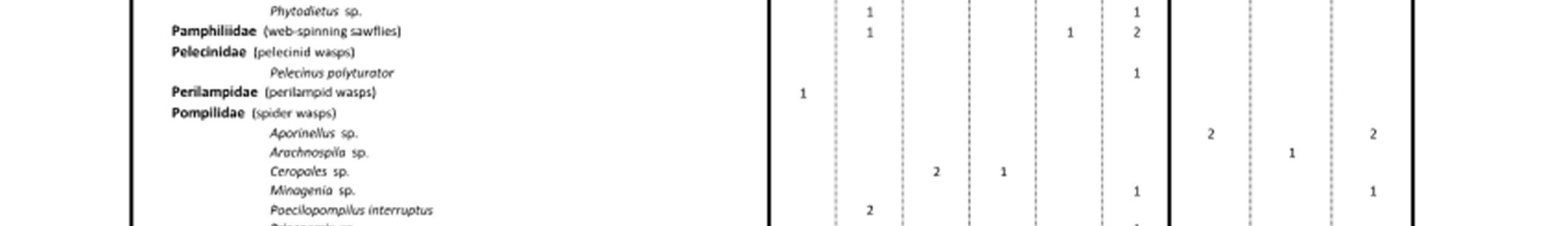
**

**S9 Table.** Comparisons between the months in which non-bee insects were collected. Bolded values are significant (P < 0.05).

| **Response Variable** | **Fixed Effect** | **Contrast** | **Random Effect** | **Effect Size** | **t value** | **p value** |
| --- | --- | --- | --- | --- | --- | --- |
| Log(Abundance) | Time, Month | AM - PM | Year, Plot | -0.23 | -1.59 | 0.11 |
|  |  | AUG - JULY |  | **1.36** | **6.62** | **< 0.001** |
|  |  | AUG - JUNE |  | **1.18** | **5.94** | **< 0.001** |
|  |  | AUG - MAY |  | **1.91** | **9.58** | **< 0.001** |
|  |  | MAY - JULY |  | **0.55** | **2.69** | **0.007** |
|  |  | MAY - JUNE |  | **0.73** | **3.64** | **< 0.001** |
|  |  | JULY - JUNE |  | 0.17 | 0.85 | 0.4 |
|  |  |  |  |  |  |  |
| Log(Species Richness) | Time, Month | AM - PM | Year, Plot | -1.22 | -1.22 | 0.22 |
|  |  | AUG - JULY |  | **4.23** | **2.95** | **0.003** |
|  |  | AUG - JUNE |  | 2.5 | 1.79 | 0.07 |
|  |  | AUG - MAY |  | **6.9** | **4.95** | **< 0.001** |
|  |  | MAY - JULY |  | 2.67 | 1.86 | 0.06 |
|  |  | MAY - JUNE |  | **-4.4** | **-3.16** | **0.002** |
|  |  | JULY - JUNE |  | 1.73 | 1.21 | 0.23 |

**S10 Table.** Results of GLMMs of treatment effects on non-bee insect abundance and morphospecies richness.

| **Response Variable** | **Fixed Effect** | **Contrast** | **Random Effect** | **Effect Size** | **t value** | **p value** | **AIC** |
| --- | --- | --- | --- | --- | --- | --- | --- |
| Log(Abundance) | **2016 application (continuous)** | **NA** | **Year, Time** | -0.001 | -1.73 | 0.08 | 68.23 |
|  | 2016 application (categorical) | LVB - HVF | Year, Time | -0.36 | -1.83 | 0.07 | 69.75 |
|  |  | LVF - HVF | Year, Time | -0.16 | -0.97 | 0.33 |  |
|  |  | LVF - LVB | Year, Time | 0.2 | 1.01 | 0.31 |  |
|  | 2016 plant species richness | NA | Year, Time | 0.008 | 0.64 | 0.52 | 70.77 |
|  |  |  |  |  |  |  |  |
| Log(Species Richness) | **2016 application (continuous)** | **NA** | **Year, Time** | < 0.001 | -0.94 | 0.35 | 54.79 |
|  | 2016 application (categorical) | LVB - HVF | Year, Time | -0.11 | 0.64 | 0.52 | 57.24 |
|  |  | LVF - HVF | Year, Time | -0.03 | -0.23 | 0.82 |  |
|  |  | LVF - LVB | Year, Time | 0.08 | 0.45 | 0.65 |  |
|  | 2016 plant species richness | NA | Year, Time | 0.003 | 0.24 | 0.81 | 55.62 |

**S1 Fig.**


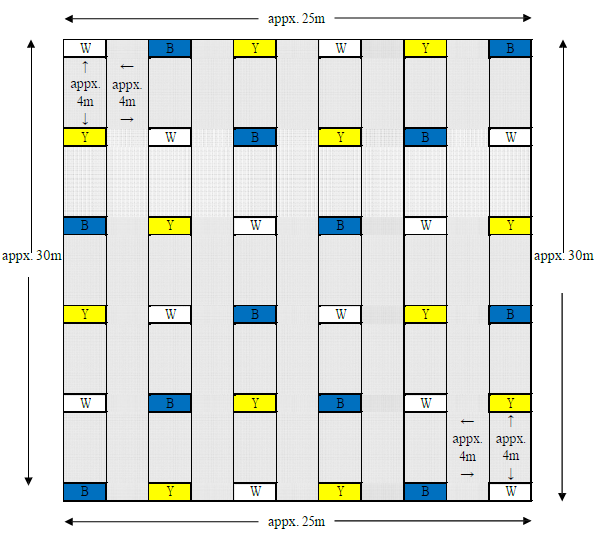


*Figure not to scale*

**S1 Fig.** Distribution of bowl traps, including white, yellow, and blue 350mL bowls set out for 24 hours once during the summer.

**S2 Fig.**

**
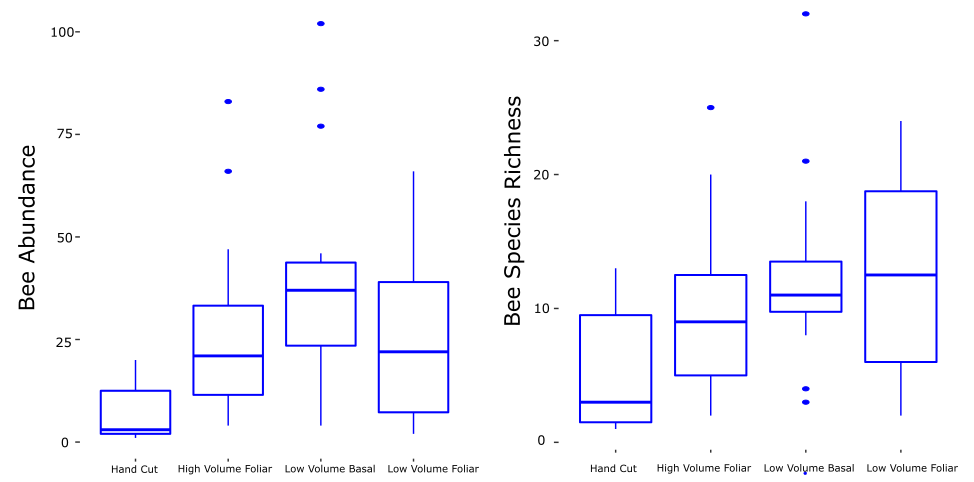
**

**S2 Fig.** Boxplots of the abundance and species richness of bees including the hand cut plots. We did not include hand cut plots in the main analysis because we were not able to sample them effectively due to a proliferation of thorny vegetation.

**S3 Fig.**


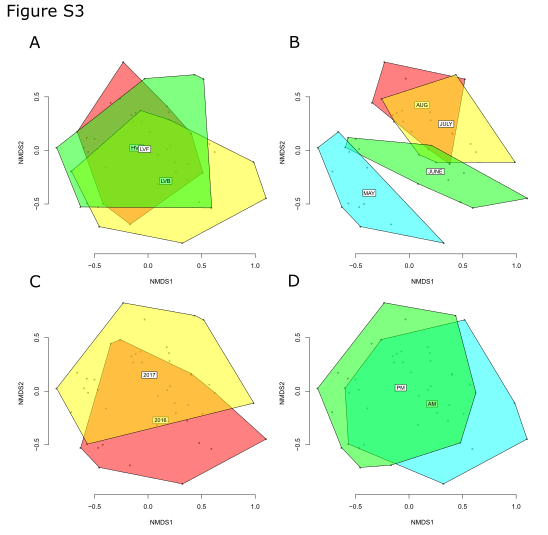


**S3 Fig.** NMDS plots showing the relationship between the bee community composition and categorical treatment (A), month of the year (B), year of the study (C), and time of the day (D). There is a lot of overlap in the community polygons except for in the month of year, suggesting that the composition of the bee community changes over the course of the year more than it does due to time of day, treatment, or between years.
